# Value of serum glycated albumin and high-sensitivity C-reactive protein levels in the prediction of presence of coronary artery disease in patients with type 2 diabetes

**DOI:** 10.1186/1475-2840-5-27

**Published:** 2006-12-20

**Authors:** Li Jin Pu, Lin Lu, Xue Wei Xu, Rui Yan Zhang, Qi Zhang, Jian Sheng Zhang, Jian Hu, Zheng Kun Yang, Feng Hua Ding, Qiu Jin Chen, Sheng Lou, Jie Shen, Dan Hong Fang, Wei Feng Shen

**Affiliations:** 1 Department of Cardiology, Institute of Cardiovascular Diseases, Rui Jin Hospital, Jiaotong Univerisity Medical School, Shanghai, People's Republic of China

## Abstract

**Background:**

Coronary artery disease (CAD) is a major vascular complication of diabetes mellitus and reveals high mortality. Up to 30% of diabetic patients with myocardial ischemia remain asymptomatic and are associated with worse prognosis compared to non-diabetic counterpart, which warrants routine screening for CAD in diabetic population. The purpose of this study was to evaluate the clinical value of serum glycated albumin and high-sensitivity C-reactive protein (hs-CRP) levels in predicting the presence of CAD in patients with type 2 diabetes.

**Methods:**

Three hundred and twenty-four patients with type 2 diabetes were divided into two groups based on presence (CAD group, n = 241) or absence (control group, n = 83) of angiographically-documented CAD (lumen diameter narrowing ≥70%). Serum levels of glycated albumin and hs-CRP as well as serum concentrations of glucose, lipids, creatinine, blood urea nitrogen and uric acid were measured in both groups. Predictors of CAD were determined using multivariate logistic regression model and receiver-operating characteristic (ROC) curves.

**Results:**

Serum glycated albumin and hs-CRP levels were significantly increased in diabetic patients with CAD. Multivariate regression analysis revealed that male gender, age, serum levels of glycated albumin, hs-CRP, creatinine and lipoprotein (a) were independent predictors for CAD. Areas under the curve of glycated albumin and hs-CRP and for regression model were 0.654 (95%CI 0.579–0.730, P < 0.001), 0.721 (95%CI 0.658–0.785, P < 0.001) and 0.824 (95% CI 0.768–0.879, P < 0.001), respectively. The optimal values of cut-off point were 18.7% (sensitivity 67.9%, specificity 60.0%) for glycated albumin and 5.2 mg/l (sensitivity 72.2%, specificity 60.0%) for hs-CRP to predict CAD. Logistic regression model was defined as: P/(1-P) = EXP(-1.5 + 1.265 gender + 0.812 age + 1.24 glycated albumin + 0.953 hs-CRP + 0.902 lipoprotein(a) + 1.918 creatinine). The optimal probability value for predicting CAD in type 2 diabetic patients was 0.648 (sensitivity 82.3%, specificity 68.6%).

**Conclusion:**

Serum glycated albumin and hs-CRP levels were significantly elevated in patients with type 2 diabetes and CAD. The logistic regression model incorporating with glycated albumin, hs-CRP and other major risk factors of atherosclerosis may be useful for screening CAD in patients with type 2 diabetes.

## Background

Coronary artery disease (CAD) is a major vascular complication in patients with type 2 diabetes. Previous studies have shown that up to 30% of the diabetic patients with CAD had silent ischemia and experienced poor outcome following acute coronary events [1], indicating clinical importance of screening asymptomatic CAD in diabetic population. Advanced glycated end products (AGEs) have been shown to form non-enzymatically in hyperglycemic environment and to be implicated in diabetic vascular complications. Recent studies indicated that AGEs-mediated inflammation is closely related to atherosclerosis process in patients with diabetes [2,3]. Glycated albumin is a predominant glycated protein in vivo and exerts adverse effects on vascular biological function. Determination of serum glycated albumin level provides more information other than assessment of glycemic control in a retrospective period [4]. Although relationship between diabetic atherogenesis and several common risk factors (e.g., hyperglycemia, hypertension and hyperlipidemia) have been established [5], few clinical studies have been made, which take glycated products into account as risk factors to predict and evaluate diabetic vascular complications. The aim of this study was to determine the clinical value of serum glycated albumin and high-sensitivity C-reactive protein (hs-CRP) levels for the prediction of CAD using logistic regression model in patients with type 2 diabetes.

## Methods

### Patients and controls

The study population consisted of 324 consecutive patients with type 2 diabetes, who underwent coronary angiography for the diagnosis and interventional treatment of CAD in the Department of Cardiology and Department of Endocrinology and Metabolic Diseases, Rui Jin Hospital, Shanghai. The diagnosis of type 2 diabetes was made according to the criteria using a fasting glucose level ≥7.0 mmol/L or 2-hour postprandial glucose level ≥11.1 mmol/L. Patients who had a clinical history of type 2 diabetes and were receiving oral hypoglycemic or parental insulin medications were also eligible for the study, but those with type 1 diabetes were excluded by measurement of serum C-peptide and insulin levels. The protocol was approved by the hospital Ethics Committee and written informed consent was obtained from all patients.

### Coronary angiography

Coronary arteriography was performed using standard Judkins techniques or through radial approach. Angiographic analysis was carried out by interventional cardiologists, who were blinded to the study protocol. Mild CAD on visual interpretation was defined as lumen diameter narrowing <50%, and significant CAD as the presence of any luminal stenosis ≥50%. For the purpose of the study, patients were divided into two groups based on visual angiographic results. Control group consisted of 83 patients without any or with mild CAD, and CAD group comprised 241 patients with ≥1 significantly affected coronary artery.

### Biochemical investigations

Blood samples were collected after overnight fasting and stored at -70°C prior to analysis. Serum total cholesterol and triglyceride levels were measured by automated enzymatic procedures (Hoffman-La Roche, Basel, Switzerland). The low-density lipoprotein cholesterol (LDL-C), high-density lipoprotein cholesterol (HDL-C) and lipoprotein (a) (Lp [a]) were determined after separating the lipoprotein fractions from fresh fasting sera by sequential ultracentrifugation. Concentration of apolipoproteins A (apo-A) and B (apo-B) were measured by immunoturbidimetric methods using commercial kit (Boehringer-Mannheim, Mannheim, Germany). Blood urea nitrogen, creatinine, and uric acid were assessed using standard methods.

Serum glycated albumin level was measured with improved bromocresol purple method using Lucica TM glycated albumin-L assay kit (Asahi Kasei Pharma, Japan). Its linear range was 3.2–68.1% and a maximum inter-assay coefficient of variation (CV) was 3.0%. The hs-CRP level was determined using a high-sensitivity ELISA kit (Biocheck Laboratories, USA) with linear range of 0.62–119.3 mg/L and inter-assay CV <7.5%.

### Statistics

All statistical analyses were performed using SPSS for Windows 13.0 (SPSS Inc., Chicago, Illinois). Data are presented as frequencies and percentages for categorical variables and mean ± SD for continuous variables, unless otherwise indicated. Differences between groups were assessed using the Chi-square and unpaired t tests. Because glycated albumin, triglycerides, hs-CRP, Lp (a), and creatinine values were not normally distributed, between-group differences were assessed by the Mann-Whitney U test. Univariate analysis was performed to determine each variable's ability in predicting CAD in patients with type 2 diabetes. Variables found to be predictive of CAD in univariate analysis were then entered into a multivariable model using logistic regression to determine the power of each variable for predicting CAD in patients with type 2 diabetes. At the last step of the analysis, the Hosmer-Lemeshow test was use to examine models' goodness of fit. The predictive values of glycated albumin and hs-CRP in the logistic regression model were calculated by constructing receiver-operating characteristic (ROC) curves. A 2-tailed P value of <0.05 was considered statistically significant.

## Results

### Baseline clinical characteristics and biochemical measurements

Baseline clinical characteristics and biochemical measurements in both groups are listed in Table 1. Patients in CAD group were older and more male gender and cigarette smoking than those in control group. Blood pressure, serum glucose level and lipid profile were similar in the two groups, except higher serum Lp (a) and reduced apo-A in CAD group. Serum blood urea nitrogen and creatinine levels were significantly higher in CAD group than in control group, indicating reduced renal function in CAD group.

**Table 1:** Baseline characteristics and biochemical assessments

**Variable**	**Control group (n = 83)**	**CAD group (n = 241)**	**P value**
Men (%)	38(45.8)	165(68.5)	<0.0001
Age (yrs)	62 ± 10	66 ± 10	0.001
Cigarette smoking (%)	11(13.3)	68(28.2)	0.007
Hypertension (%)	52(62.7)	175(72.6)	NS
Blood pressure			
Systolic (mmHg)	138 ± 21	137 ± 20	NS
Diastolic (mmHg)	81 ± 10	79 ± 11	NS
Hyperlipidemia (%)	47(56.6)	137(56.8)	NS
Cholesterol			
Total cholesterol (mmol/L)	4.77 ± 1.27	4.70 ± 1.15	NS
HDL-cholesterol (mmol/L)	1.19 ± 0.30	1.13 ± 0.41	NS
LDL-cholesterol (mmol/L)	2.80 ± 0.89	2.73 ± 0.90	NS
Triglycerides(mmol/L)	2.23 ± 1.79	2.01 ± 1.19	NS
Lipoprotein-a (g/L)	0.19 ± 0.11	0.25 ± 0.19	0.025
Apo-A (g/L)	1.29 ± 0.19	1.24 ± 0.22	NS
Apo-B (g/L)	0.94 ± 0.26	0.94 ± 0.25	NS
Glucose (mmol/L)	7.03 ± 1.88	7.28 ± 2.72	NS
Blood urea nitrogen (mmol/L)	5.49 ± 1.60	6.05 ± 2.29	0.043
Creatinine (μmol/L)	74.67 ± 16.31	92.59 ± 29.22	<0.0001
Uric acid (μmol/L)	307.9 ± 76.03	314.19 ± 83.07	NS
Glycated albumin (%)	19.37 ± 4.29	21.21 ± 5.24	0.001
Hs-CRP (mg/L)	7.37 ± 8.71	25.77 ± 30.35	<0.0001

### Logistic regression model

Serum glycated albumin and hs-CRP levels were significantly increased in diabetic patients with CAD than in controls (Table 1). Multivariate regression analysis revealed that older age, male gender, serum concentrations of glycated albumin, hs-CRP, Lp (a), and creatinine were independent risk factors for CAD development in patients with type 2 diabetes (Tables 2 and 3). The logistic regression model for predicting CAD in diabetes was defined as: P/(1-P) = EXP(-1.5 + 1.265 gender + 0.812 age + 1.24 GA + 0.953 hs-CRP + 0.902 Lp[a] + 1.918 creatinine), and the probability value for each patient was then calculated by equation: P = e^y^/1+e^y^, where y = ln [P/(1-P)]. Hosmer-Lemeshow test was used to check the models' goodness of fit and the results demonstrated a good fit achieved (goodness of fit: Hosmer-Lemeshow *χ*^2 ^= 7.465, d.f. = 8, P = 0.487). Nagelkerke R^2 ^value was 0.35, suggesting the model explained 35% of the variation in the dependent variable.

**Table 2:** Univariate predicators of coronary artery disease in patients with type 2 diabetes

**Variable**	**Odds Ratio**	**95% Confidence interval**	**P Value**
Men	3.573	1.64–7.81	0.001
Age ≥ 65 Yrs	2.267	1.12–4.57	0.022
Cigarette smoking	1.026	0.4–2.63	0.958
Hypertension	1.158	0.57–2.36	0.686
Hyperlipidemia	1.252	0.59–2.64	0.556
Total cholesterol	1.139	0.53–2.44	0.737
HDL-cholesterol	0.871	0.57–1.34	0.568
LDL-cholesterol	0.937	0.38–2.29	0.887
Triglycerides	1.017	0.71–1.46	0.927
Lipoprotein-a ≥ 0.22 g/L	2.057	1.03–4.09	0.04
Blood urea nitrogen	0.955	0.80–1.15	0.624
Creatinine ≥ 100 (μmol/L)	8.109	2.06–31.9	0.003
Uric acid	1	1.00–1.01	0.849
Glycated albumin ≥ 20%	3.54	1.74–7.20	<0.001
Hs-CRP ≥ 10 mg/L	2.648	1.32–5.30	0.006

**Table 3:** Multivariate predictors of coronary artery disease in patients with type 2 diabetes

**Variable**	**B**	**Odds ratio**	**95% CI**	**P value**
Constant	-1.5	0.223		<0.0001
Men	1.265	3.542	1.818–6.689	<0.0001
Age ≥ 65 Yrs	0.812	2.253	1.161–4.372	0.016
Glycated albumin ≥ 20%	1.24	3.456	1.777–6.720	<0.0001
Hs-CRP ≥ 10 (mg/L)	0.953	2.593	1.313–5.119	0.006
Lp (a) ≥ 0.22 g/L	0.902	2.464	1.249–4.858	0.009
Creatinine ≥ 100 umol/L	1.918	6.805	1.956–23.673	0.003

### Receiver-operating characteristic curves

**Figure 1:**
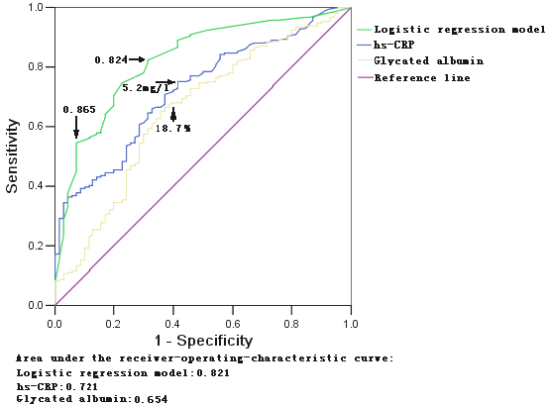
ROC curve for glycated albumin, hs-CRP and logistic regression model for predicting coronary artery disease in patients with type 2 diabetes.

ROC plot was calculated to test predictive value of glycated albumin and hs-CRP, and the effectiveness of logistic regression model was also evaluated by constructing ROC plot. A larger area under the curve of hs-CRP was observed compared to glycated albumin. The optimal value of cut-off point for hs-CRP and probability value of regression model to predict CAD in patients with type 2 diabetes were 5.2 mg/l and 0.648, respectively. When using hs-CRP as a single predictor, 67 out of 241 diabetic patients with CAD (27.8%) could be missed (sensitivity 72.2%, specificity 60.0%). The use of regression model may cause 43 out of 241 CAD cases (17.7%) missed (sensitivity 82.3%, specificity 68.6%). The ROC plot showed that the optimal cut-off point of regression model for diagnosis of CAD was 0.865, with only 6 out of 83 cases (7.1%) without CAD being falsely recognized as CAD (sensitivity 54.5%, specificity 92.9%) (Figure 1, Table 4).

**Table 4:** Diagnostic performance of glycated albumin, hs-CRP and logistic regression model

**Variable**	**Area under curve (95% CI)**	**P value**	**Cut-off point**	**Sensitivity**	**Specificity**
Glycated albumin	0.654(0.579–0.730)	<0.0001	18.7%	67.9%	60%
Hs-CRP	0.721(0.658–0.785)	<0.0001	5.2 mg/L	72.2%	60%
Regression model	0.824(0.768–0.879)	<0.0001	0.648	82.3%	68.6%

## Discussion

Diabetes has been recognized as an important risk factor for CAD, and diabetic patients are at 2-fold increased risk of cardiovascular mortality compared to their nondiabetic counterparts [6]. Previous studies have demonstrated that silent myocardial ischemia, which is mainly caused by autonomic neural dysfunction, occurred in about 20% to 25% of diabetic patients, and the prevalence may be as high as 60% in those at high-risk [7]. Because silent myocardial ischemia is responsible for more delayed diagnosis of CAD and poorer prognosis than anginal episodes, early detection and routine screening of CAD with simple tool becomes important and desirable for diabetic population [1,6,8]. The current study indicates that several serum biomarkers could be predictive for CAD in patients with type 2 diabetes, among which serum levels of hs-CRP and glycated albumin appeared to be useful in clinical practice.

### Serum Lp(a) concentration is an independent risk factor for CAD in patients with type 2 diabetes

Numerous studies have shown that elevated serum level of Lp (a) is an independent risk factor for CAD in nondiabetic subjects, but its contributions to atherosclerosis in diabetes remains in controversy [9-12]. In the present study, Lp (a) was found to be an independent risk factor for CAD development in a diabetic cohort, while HDL-C, LDL-C, total cholesterol, triglyceride, hypertension, and cigarette smoking did not enter this model, suggesting that these traditional risk factors were not risk factors for CAD development in these diabetic subjects. However, we do not believe that the impact of these risk factors can be excluded because of their being quantitatively not different from those in control group or within normal range, compared to Lp(a). They may exert detrimental effects depending on how much they become glycated and oxidized, which is not easy to determine at present. Cumulative evidence indicates that qualitative changes in lipoproteins may contribute to diabetic angiopathy. Glycated and/or oxidized LDL have demonstrated adverse effects on vascular cell viability, lipid accumulation, growth factor expression and intracellular oxidative stress [13]. Modification of HDL in diabetes by glycation and oxidation may ameliorate efficacy of its vasoprotective functions [13]. In addition, majority of the diabetic patients with CAD in our study received medication of statins, and Lp (a) is less sensitive to statin treatment, compared to other lipoproteins. This may partly explain why Lp(a) level was higher and entered the model. In this study, serum creatinine level was significantly related to the presence of CAD, and this is consistent with previous findings that patients with renal dysfunction had high incidence of cardiovascular disease, particular in those with late stage renal insufficiency [14,15].

### Serum glycated albumin and hs-CRP predict CAD development in patients with type 2 diabetes

Glycated albumin is a kind of early-stage amadori-modified reaction products formed from Schiff's base adducts [16], and has been implicated in the pathogenesis of diabetic complications [17,18]. Previous studies have demonstrated that glycated albumin induces oxidative stress in the vessel wall [19], enhances pro-inflammatory endothelial response to S100A8/A9 [20], and promotes proliferation and migration of vascular smooth muscle cells [21], and thereby is associated with accelerated atherosclerosis. The present study showed that glycated albumin was an independent risk factor for CAD in patients with type 2 diabetes, with odds ratio being 3.456(95% CI 1.78–6.72, P < 0.001).

Measurement of blood HbA1c level has been widely used to evaluate glycemic control in diabetic patients. We previously observed a significant correlation between serum glycated albumin level and HbA1c concentration (r = 0.743, P < 0.001, unpublished data). Previous studies have further shown that serum glycated albumin level plateaued several weeks in diabetic patients in whom serum glucose levels were initially poorly controlled and then intensive insulin management was used to rapidly bring glucose levels under control, while serum HbA1c levels were still falling [6]. Therefore, these results indicate that determination of serum glycated albumin level appears to be a useful marker for predicting the presence of diabetic complications, and also a valuable adjunct to HbA1c measurement in reflecting short-term glycemic control.

Atherosclerosis is a long-term and chronic inflammatory process that is exacerbated in patients with diabetes [22,23]. CRP has been shown to be associated with arteriosclerosis and acute cardiovascular events [24], and has been widely used to stratify patients at high-risk for acute coronary events. Researches showed that patients at intermediate or high risk of CAD may benefit from measurement of hs-CRP with regard to individual risk prediction [25]. The current study revealed that serum level of hs-CRP ≥ 10 mg/L was associated with a 2.593-fold increase in risk of CAD in type 2 diabetes when compared with hs-CRP level < 10 mg/L. It has been found that good glycemic control per se did not affect nontraditional risk factors for CAD equally despite improved serum glucose level [26], and increased hs-CRP levels were associated with other indicators of diabetes-related cardiovascular risk, but had no correlation with disease duration or glucose control [27,28]. These results indicates that serum levels of hs-CRP reflect inflammatory status in type 2 diabetes regardless of glycemic control, which merits hs-CRP to be an independent biomarker for predicting CAD. Aggressive intervention of AGEs and inflammatory process is crucial for preventing macrovascular complications in patients with type 2 diabetes, and many agents have been shown to reduce hs-CRP concentrations and to inhibit AGEs formation in these patients [22,25,29].

### Predictive value of logistic regression model

Another major finding of the present study was that the use of logistic regression model incorporating with glycated albumin, hs-CRP and other major independent risk factors commonly seen in type 2 diabetes was competent to screen and predict CAD in patients with diabetes (area under curve: 0.824). The optimal probability value of 0.648 may be more suitable for screening CAD in this cohort with a sensitivity of 82.3% and a specificity of 68.6%. Furthermore, the probability value 0.865 was particularly useful because of its high specificity (92.9%). Previous study showed that positive predictive value of ECG exercise stress test for angiographically-documented CAD in patients with diabetes was 73% [6]. Our results suggest that the regression mode may be as effective as exercise stress tests for screening CAD in clinical practice with low cost, and it could also provide useful information regarding the evaluation of the effects of therapeutic modalities aiming to reduce incidence of cardiovascular events in type 2 diabetes.

### Limitations

The present study has several limitations. First, this is in fact a retrospective study and the sample size in the control group was relatively small. Further study is required to increase sample size to ascertain the differences in biomarkers and biochemical measurements between diabetic patients with CAD and controls. As follow-up of the patients has been undergoing and therefore our logistic regression model of CAD prediction in diabetes could be tested by clinical data regarding patient's outcome and disease progression. Secondly, hs-CRP was the only inflammatory factor measured in this study. Whether other "noxious" pro-atherosclerosis inflammatory factors, such as interleukin-6, play a role in the development of CAD in this cohort remains to be answered. As suggested in some studies, a cluster of "harmful" interleukins exert synergistic and convergent effects on cardiovascular diseases and glucose metabolic alterations [30]. Determination of interleukin helps understand the mechanisms of cardiovascular diabetology on disease progression and thereby provides potential guidance to simultaneously control both atherosclerosis and diabetes. Finally, the Nagelkerke R^2 ^value of the regression model was only 0.350, suggesting that the model only explained 35% of variation in the dependent variable, which leaves space for some other pro-atherosclerosis risk factors to complete. Among these possible factors, soluble receptor of AGEs and other factors associated with AGE-receptor system may warrant further investigation.

## Conclusion

Serum glycated albumin and hs-CRP levels were elevated in diabetic patients with CAD. The logistic regression model incorporating with glycated albumin, hs-CRP and other major risk factors of atherosclerosis may be useful for screening CAD in patients with type 2 diabetes.

## Abbreviations

High-density lipoprotein (HDL), low-density lipoprotein (LDL); lipoprotein a [Lp (a)]

## Competing interests

The author(s) declare that they have no competing interests.

## Authors' contributions

LJP collected the samples and performed the experiments, LL designed the study and participated in the whole process of experiments; XWX, RYZ, QZ, JSZ, JH, ZKYang, FHD, QJC, SL, JS and DHF participated or were partly involved in this work. WFS was responsible for the whole research project and was involved in the whole process of the study in its design and coordination, and preparation of the manuscript. All authors read and approved the final manuscript.
